# Situation of ophthalmology education in Brazil: supply versus demand

**DOI:** 10.5935/0004-2749.2021-0482

**Published:** 2023-09-27

**Authors:** Newton Kara-Junior, Rafael Scherer, Camila Koch, Paulo Augusto de Arruda Mello

**Affiliations:** 1 Ophthalmology Department, Universidade de São Paulo, São Paulo, SP, Brazil; 2 Ophthalmology Department, Universidade Federal de São Paulo, São Paulo, SP, Brazil

**Keywords:** Ophthalmology, Teaching, Medical education, Specialization, Oftalmologia, Ensino, Educação médica, Especialização

## Abstract

**Purpose:**

The number of medical schools in Brazil has increased in recent years; however, vacancies for specialization in ophthalmology probably have not kept up with the growing demand. This study wants to estimate the increase in medical schools, the demand for ophthalmology specialization, and evaluate learning opportunities in ophthalmology.

**Methods:**

This was a retrospective study with research from the Ministry of Education and Brazilian Council of Ophthalmology database from 2002 to 2021. These data were checked through 120 notices published by the institutions in 2021.

**Results:**

The number of medical school vacancies increased by 370%, whereas the number of certified ophthalmology vacancies increased by 64%. There was an 11.4% misalignment between the Brazilian Council of Ophthalmology data in the Ministry of Education.

**Conclusion:**

The proportion of medical graduates has increased much more than opportunities for ophthalmology specialization. The effect on the search for unaccredited specialization positions is unknown, and policies for monitoring the specialization of ophthalmology vacancies should be established.

## Abstract (pt)

**Objetivo:**

Nos últimos 20 anos, o número de escolas médicas no Brasil aumentou, mas as vagas para especialização em Oftalmologia não acompanharam a demanda crescente. Este estudo quer estimar a demanda por especialização e avaliar a oferta de oportunidades de aprendizado em Oftalmologia.

**Métodos:**

Estudo epidemiológico com pesquisa em banco de dados provenientes do Ministério da Educação e Conselho Brasileiro de Oftalmologia. Estes dados foram checados através de 120 editais publicados pelos serviços de Residência em 2021.

**Resultados:**

De 2002 a 2021, o número de vagas em faculdades de Medicina aumentou 370%, enquanto o número de vagas certificadas de especialização em Oftalmologia aumentou 64%. Houve um desalinhamento de 11.4% entre os dados do Conselho Brasileiro de Oftalmologia e do Ministério da Educação.

**Conclusão:**

A proporção de graduados em Medicina aumentou muito mais do que a oferta de oportunidades de especialização em Oftalmologia, o impacto disto na busca por vagas de especialização não acreditadas é desconhecido, políticas de monitoramento das vagas de especialização em Oftalmologia devem ser estabelecidas.

## INTRODUCTION

In the past 20 years, the number of medical schools in Brazil has increased. However, the vacancies for specialization in ophthalmology have probably not kept pace with the increased demand, especially in better-structured university services, which in some cases even reduced the number of residents because of budgetary crises that limited the number of procedures^(1,2,3)^.

Official vacancies for training in the specialty of ophthalmology in Brazil are registered with the *Conselho Brasileiro de Oftalmologia* (CBO) and at the Ministry of Education and Culture (MEC). However, some physicians who are unable to access official ophthalmology vacancies are willing to undertake training in ophthalmology in noncertified services. In these services, teaching requirements are not regulated unlike programs accredited by the CBO or MEC, which have mandatory requirements.

This article aimed to estimate the number of educational opportunities in ophthalmology in Brazil using data published from 2002 to 2021, characterize training in the specialty, and produce a guide for educational policies.

## METHODS

### Data collection

A retrospective study was performed by searching databases for information regarding the number of medical schools and the number of ophthalmology vacancies in Brazil from 2002 to 2021.

Medical school vacancies

Data were collected from the National Institute for Educational Studies and Research Anísio Teixeira (INEP) (Microdata Higher Education Census 2019), linked to the National Commission of Medical Residencies (CNRM) of the MEC.

### Ophthalmology vacancies

Data registered by the MEC regarding the number of ophthalmology residency vacancies in this period were analyzed. A database containing the number of ophthalmology vacancies accredited by the CBO was also examined.

The 2021 data were checked through medical residency application notices published by the ophthalmologic institutions. Moreover, two emails were sent to the residency program coordinators or they were called to verify the data.

Data sources, as well as how they were accessed, are highlighted in the bibliographic references^(1,2,4)^.

### Statistical analysis

Quantitative variables are expressed as the mean and standard deviation. Quantitative variables were expressed by absolute and relative frequencies. There was no need for sample validation because all data were used.

## RESULTS

### Historical medical and ophthalmology vacancies

The number of medical school vacancies increased by 370%, i.e., from 11,243 in 2002 to 52,873 in 2019 (Table 1), whereas the number of CBO-certified ophthalmology vacancies increased by 64%, i.e., from 286 and 319 in 2002 to 495 (+73%) and 498 (+56%) in 2019, according to MEC and CBO, respectively (Table 2).

**Table 1:** Number of medical school vacancies per year in Brazil

Year	Medical school vacancies (INEP)
2002	11.243
2003	12.281
2004	14.102
2005	14.661
2006	15.278
2007	16.241
2008	17.504
2009	16.876
2010	16.468
2011	16.752
2012	17.515
2013	18.574
2014	22.787
2015	24.302
2016	27.346
2017	31.025
2018	48.571
2019	52.873
2020	NA
2021	NA

INEP= National Institute for Educational Studies and ResearchNA= Not available.

**Table 2:** Total number of ophthalmology R1 residency vacancies in Brazil according to the accreditation institution

	Ophthalmology positions	
Year	MEC	CBO
2002	286	319
2003	314	NA
2004	322	NA
2005	324	322
2006	318	310
2007	316	310
2008	325	307
2009	332	305
2010	NA	298
2011	NA	330
2012	NA	330
2013	404	343
2014	455	393
2015	502	435
2016	499	438
2017	509	482
2018	530	487
2019	495	498
2020	483	NA
2021	481	489

CBO= Brazilian Council of Ophthalmology; MEC= National Commission of Medical Residencies of the Ministry of Education; NA= not available.

### Audit of vacancies reported by CNRM and CBO in 2021

The 2021 data were determined through 120 medical residency application notices published by the ophthalmologic institutions, which accounted for 78.5% of the vacancies posted that year.

A second check was conducted via phone and email, which confirmed 25.3% of the services (Table 3). A total of 74.7% of vacancies could not be confirmed because services did not respond to calls and 1.3% refused to respond.

**Table 3:** Audit by phone and email of vacancies reported by CNRM and CBO in 2021

Contact details with institutions	n	%
Data as reported by CNRM/CBO	17	10.8
Data in disagreement with CNRM/CBO	18	11.4
No reply	118	74.7
No medical residency in ophthalmology	3	1.9
Refused to answer	2	1.3
**Total contacted**	**158**	**-**

CBO= Brazilian Council of Ophthalmology; CRNM= National Commission of Medical Residencies of the Ministry of Education.

### R1 ophthalmology vacancies by region in 2021

Positions validated by the audit were divided by macro regions and compared with the IBGE Census in 2020, with a discrepant ratio of vacancies per inhabitant in the north and northeast regions versus the southeast and south regions (Table 4).

**Table 4:** R1 ophthalmology vacancies by region in 2021

Region	CBO	CNRM	Total	% From total	Institutions	Population (IBGE 2020)	Vacancies per 1 MI inhabitants
N	26	17	31	5.0	8	18.672.591	1.7
NE	95	94	119	19.1	31	57.374.243	2.1
CW	30	42	49	7.9	15	16.504.303	3.0
SE	257	226	321	51.6	76	89.012.240	3.6
S	69	77	102	16.4	24	30.192.315	3.4
**Total**	**477**	**456**	**622**	-	**154**		

CBO= Brazilian Council of Ophthalmology; CRNM= National Commission of Medical Residencies of the Ministry of Education; IBGE= Brazilian Institute of Geography and Statistics

### General data on R1 ophthalmology vacancies in 2021

The data audit showed that a total of 153 institutions registered 622 residency positions in ophthalmology in 2021,477 were registered with CBO and 456 with CNRM, and 50% of the slots were registered with both. The CRNM keeps records of vacant vacancies, reporting 25 unoccupied R1 vacancies (4% of the total accredited positions in the country) (Table 5).

**Table 5:** General data on 2021 R1 vacancies

General data	n	%	Total
*Institutions*	153*	-	-
Total vacancies in Brazil	622	-	-
*Total CBO vacancies*	477**	-	-
*Total CNRM vacancies*	456**	-	-
*Dually accredited vacancies*	311	50%	622
*Total CRNM R1 idle vacancies*	25	4%	622

CBO= Brazilian Council of Ophthalmology; CRNM= National Commission of Medical Residencies of the Ministry of Education* Five of 158 institutions reported no vacancies for ophthalmology.

** These data were corrected based on public notices and contact with institutions.

## DISCUSSION

The creation of the medical residency programs in the country was made official in 1977. Since CBO was founded in 1941, it has progressively strengthened and standardized ophthalmic training. Additionally, the medical residency service, a public institution, offers ophthalmology training accredited by the CNRM of the MEC(^5,6^).

Although the number of ophthalmologists needed in Brazil is not exactly known, no evidence shows that there has ever been a surplus of professionals. Based on 2015 data, Resnikoff et al.^(7)^ reported a prevalence of approximately 1 in 270 thousand inhabitants in underdeveloped countries versus 1 in 13 thousand in developed countries. They found a weak correlation between the density of ophthalmologists and the prevalence of blindness; thus, clearly, other factors in the patient care journey influence this morbidity.

The Brazilian Council of Ophthalmology, based on WHO recommendations, considers adequate rates between 1 for 17 thousand and 1 for 18 thousand inhabitants^(8)^. The medical assessment in 2021 conducted by this institution found values of 1 ophthalmologist for every 10,875 inhabitants in the country, a score considered adequate.

This study showed that the medical education “bottle neck” was probably overcame; however, if more ophthalmology specialists are needed, the current limitation is the ability to offer specialized training. To return to the 2002 ratio, with 35.2 undergraduate medical students for every CBO ophthalmology slot (319 CBO vacancies divided by 11,243 undergraduate medical vacancies) (Table 1), creating 1,002 new CBO specialization slots (52,873 undergraduate medical slots in 2019 divided by 35.2 minus 498 CBO vacancies in 2019) would be necessary. Santos Júnior et al.^(9)^ surveyed new medicine courses in the country and related their data to a predominantly private expansion in undergraduate medical vacancies, poor distribution, in addition to having minimum indicators for their maintenance according to the National Performance Exam of Students^(10,11,12)^. The current review measures the imbalance between the offer of specialization vacancies in ophthalmology and the professionals qualified for their claim. Therefore, evaluating the effect on the search for non-accredited specialization positions will be possible.

A discrepancy was noted in the number of vacancies open by region of the country in relation to its population (Table 4), where the ratio of certified vacancies was 1.7 per 1 million inhabitants in the north versus 3.6 in the southeast. This is consistent with the indices found by the 2021 Census of the CBO^(8,13)^, where the region with the lowest density of ophthalmologists was in the north (1: 19,512) versus the southeast region with a higher rate (1: 7,843). The effect of the decentralization of medical residency programs on the establishment of professionals in these regions should be discussed^(14)^.

In addition, in 2021, only 50% of the vacancies were accredited by both governing bodies, which should be better analyzed to investigate the causes of this mis match between MEC and CBO (Table 5). Moreover, factors related to this value may be related to the option of services to increase the number of vacancies without overlapping them or reduction in the cost related to the provision of a residency scholarship in the case of the MEC modality, among others.

The regulation of ophthalmology education services follows strict criteria, with the MEC and CBO requiring professors with academic titles, ties with the public health service, regimental structure, and infrastructure defined by CBO Bylaws or Resolution 02/2006 of the MEC. These demanding criteria make it possible to train ophthalmologists who are better prepared to treat patients. However, the strictness of the criteria may be one of the reasons why the number of specialization vacancies in ophthalmology failed to keep up with the number of new medical vacancies in Brazil in recent years.

The high demand from newly graduated doctors for specialization in ophthalmology is evident. One of the largest public notices for medical residency in the country, SUS-SP, which offers 14 positions in several hospitals, had a list of 45 candidates per position in 2018, and listed ophthalmology as the third largest specialty sought^(15)^. Given that the supply of accredited residencies is less than the demand, there are unofficial residencies in Brazil. These health services, without the education structure required by MECs or CBOs, do not have the “official quality seal” for the training of professionals.

The most critical issue is not whether the number of ophthalmologists trained annually in Brazil is adequate but whether the technical quality of doctors is adequate because of the number of medical graduates exceeding the number of formal opportunities for specialization. The National Ophthalmology Exam (PNO), title of Specialist in Ophthalmology granted by the Brazilian Medical Association, allows the professional to be recognized as a specialist if approved after the training period. Data on the pass rates in the PNO in 2018 (Table 6) indicate higher rates among physicians from an accredited course (66.22%) than among those from non-accredited courses (32.4%), which suggests that, in the past decade, there are more technically unprepared doctors working in ophthalmology.

**Table 6:** Enrolled and approved in the 2018 National Ophthalmology Test

		Approved	
Origin	Enrolled	n	%
*Accredited*	595	394	66.22
*Non-accredited*	139	58	32.4
*Non-approved (last years)*	23	6	30
**Total**	**757**	**458**	-

CBO= Brazilian Council of Ophthalmology; CRNM= National Commission of Medical Residencies of the Ministry of Education.

Owing to the lack of registration of these unaccredited vacancies, which we call independent vacancies, it is difficult to account for the total number of ophthalmologic vacancies in Brazil. Table 5 reveals an 11.4% disagreement between the data reported by the CBO, MEC, and institutions, which reinforces the challenge of verifying the number of open positions for ophthalmology specialists in the country.

The limitations of this study suggest that the MEC and CBO accreditation services must be better aligned, and the process of open vacancies should be continuously monitored. To create policies for monitoring physician training, we first need to know precisely how many there are.
